# Supplementary material for: Monolithic thin-film lithium niobate broadband spectrometer with one nanometre resolution
Source: Nat Commun. 2024 Mar 14;15:2330. doi: 10.1038/s41467-024-46512-4 (PMC10940581; doi:10.1038/s41467-024-46512-4)
Supplement: Supplementary file 1 — Supplementary Information [file 41467_2024_46512_MOESM1_ESM.pdf]

Supplementary material: Monolithic thin-film lithium niobate broadband spectrometer with one nanometre resolution

Giovanni Finco,* Gaoyuan Li, David Pohl, Marc Reig Escalé, Andreas Maeder, Fabian Kaufmann, and Rachel Grange

ETH Zurich, Department of Physics, Institute for Quantum Electronics, Optical Nanomaterial Group, Auguste-Piccard-Hof, 1, 8093, Zurich, Switzerland

(Dated: February 9, 2024)

- **Note 1: Device efficiency**
- **Note 2: DC drift and data analysis**
- **Note 3: Measurement of broadband sources**
- **Note 4: Bandwidth limitations and multi-mode operation**

*gfinco@phys.ethz.ch

NOTE 1: DEVICE EFFICIENCY

In the context of SWIFTSs, the device efficiency is calculated by considering the fraction of guided energy that is scattered out by EFSs and participates to the formed interferogram. This quantity can be derived theoretically and is calculated as

$$\eta = 2\kappa L \cdot \exp\left(-\kappa L\right), \tag{1}$$

where κ is the linear attenuation factor due to scattering of the evanescent field, and L is the sampling region length [1]. The calculation assumes a continuous medium, i.e. a standing wave that is continuously sampled across the waveguide, while for practical implementations sampling happens in a discrete fashion. Therefore, κ represents the scattering efficiency of each EFS, while L is the number of nanosamplers. The internal efficiency of SWIFTS devices can be thus maximised by tailoring the scattering efficiency of EFSs in order to optimise the extracted power from the guided mode that contributes to building up the interferogram. The internal efficiency (1) peaks at value of κ correspondent to $\kappa L = 1$, meaning that a device with optimal efficiency gradually scatters the whole energy out to build the interferogram: given an EFS number N, each nanowire should scatter a portion $1/N$ of the energy out to the detector; with our design and current experimental setup, the optimum scattering efficiency is $\kappa_{MAX} = 1.2\%/\text{wire}$ (or 0.052 dB/wire).

Another fundamental constraints exists, however, when designing EFSs for this kind of devices, and the fundamental limitation resides on the physical size of such nanosamplers compared to the target wavelength. In order to be reasonably assumed point samplers, the nanowires width (along the mode propagation direction) needs to be sufficiently small compared to the standing wave period, that in the context of waveguides can be calculated as

$$\Lambda = \frac{\lambda}{2n_{eff}}, \tag{2}$$

and corresponds to approximately $\Lambda = 400\,\text{nm}$ at 1550 nm on our platform. We therefore choose to pattern our nanowires with a width of 1/8 the standing wave period, i.e., $w_s = 50\,\text{nm}$ is the design width of our EFSs in order for them to act as point samplers.

We performed finite-difference time-domain (FDTD, Ansys Lumerical) simulations of our scattering waveguides in order to first understand whether the new platform would introduce complications in the working principle of the device due to, e.g., stronger light confinement and larger overlap of the mode with EFSs, compared to the hybrid SiN-LN version. Second, we used the simulation tool to design nanowires that would enhance the internal efficiency of our devices towards an optimal value. Due to memory constraints, a full 3D FDTD simulation could not be run for the whole extent of the sampling waveguide, so the domain size was reduced to 70 µm, for a total of 22 nanowires. Figure S1 displays the design procedure results, with the main plot reporting the normalised power decay as recorded with a line monitor placed inside the waveguide and an exponential fit to the data (orange line) from which the scattering efficiency was calculated to $\kappa = 1.1\ \%/\text{wire}$. Being the value close to the optimal efficiency, the simulated parameters (metal thickness $t_s = 17.5\,\text{nm}$, width $w_s = 50\,\text{nm}$) were chosen as main design parameters to then experimentally sweep around.

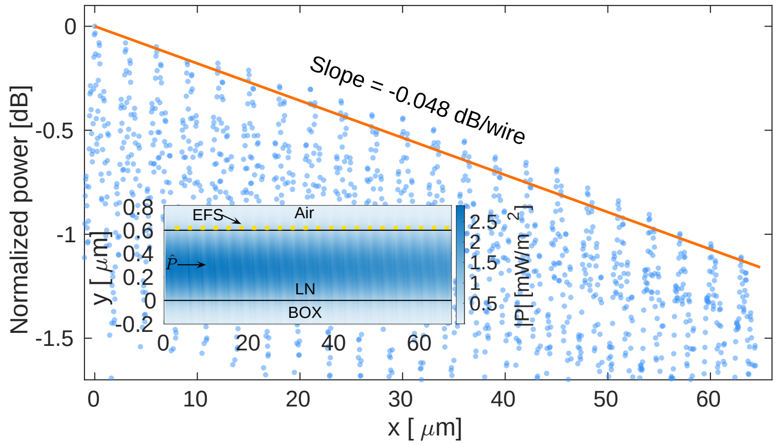

Figure S1: **FDTD simulation of scattering waveguide.** Normalised power trace recorded with a line monitor placed inside the waveguide. The orange line shows the result of an exponential fit to the data-points ($R^2 = 0.996$) from which a scattering efficiency of $-0.048\,\text{dB/wire}$ (1.1%/wire) was extracted. The inset shows the power profile inside the waveguide as calculated with a 2D FDTD simulation of an equivalent slab waveguide. Nanowires parameters are: width $w_s = 50\,\text{nm}$, thickness $t_s = 17.5\,\text{nm}$, infinite length (extending through the perfectly matched layers), and the simulation material is bulk gold.

The inset of Fig. S1 shows the power profile (Poynting vector) inside the waveguide as calculated with a 2D FDTD simulation. Each scattering event causes a partial back-reflection of the travelling energy that forces the mode to interfere

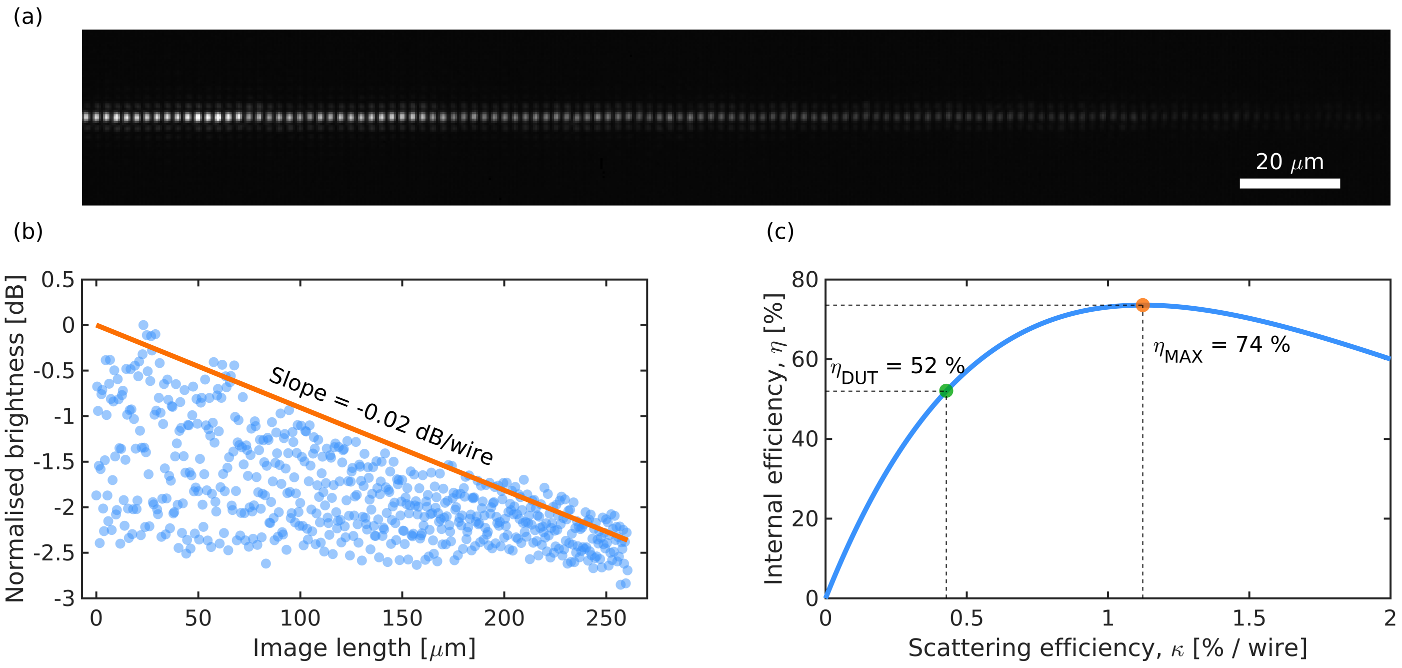

Figure S2: **Characterisation of EFS.** (a) IR image of an array of nanowires fabricated onto an LN waveguide, the pattern is generated by an optical mode propagating in only one direction to observe the intensity exponential decay at 1550 nm. (b) Normalised brightness of the image, where the orange line shows an exponential fit to the pixel intensity envelope. (c) Calculated internal device efficiency, where the solid line displays the efficiency curve for a SWIFTS with our design parameters; device (η_{DUT}) and maximum (η_{MAX}) efficiencies are highlighted with green and orange markers, respectively. EFSs parameters are as described in the main text.

with itself. Further EFS optimisation would require spacing the nanowires from the waveguide with, e.g. a thin oxide layer, in order to reduce disturbances introduced to the travelling wave and carefully tailor their scattering strength. Incidentally, numerical simulations show that the most relevant parameter to control the scattering strength is the metal thickness rather than its width.

Scattering nanowires were then fabricated on LNOI waveguides with the same parameters as described in the main text. Light was edge-coupled to the waveguide in a single propagation direction, i.e., without recombining the two signals in order to avoid the establishment of a standing wave. The scattering efficiency of fabricated EFSs was then measured by taking IR images with the same experimental setup as used to conduct spectrometry experiments, and the power decay introduced by the samplers was estimated by measuring the exponential brightness decay along the scattering waveguide as illustrated in Fig. S2. Figure S2(a) shows a sample IR image used to estimate the scattering efficiency, while S2(b) displays the normalised image brightness as obtained by summing each column of the picture down, with the orange line representing an exponential fit to the data-points envelope. Among the swept parameters, the best performing EFSs were identified for a thickness $t_s = 20\,\text{nm}$ as reported in the diagrams, from which the scattering efficiency was obtained as the slope of the curve in logarithmic scale to $\kappa = -0.02\,\text{dB/wire}$ (or 0.43 %/wire of the evanescent field energy). This translates into an internal device efficiency of $\eta_{DUT} = 52\%$ (optimal being $\eta_{MAX} = 74\%$) by considering 89 nanowires patterned with a 3 μm spacing on our spectrometer devices recombination region as displayed in Fig. S2(c). Figure S2(a) was obtained from an array of EFS with only 2 μm spacing. We increased such a distance to avoid potential cross-talk when changing the signal wavelength during spectrometry experiments. The measured efficiency is lower than the target simulated value by a factor slightly higher than two: this can be attributed to several aspects, among the most important are fabrication imperfections (fine control of the metal thickness is very hard for such a small structures) and difference between the simulated and fabricated material constants, which incidentally do not include the thin chromium layer used for adhesion. As previously mentioned, increasing the nanowires thickness will enhance the scattering efficiency, yet also introduce a higher degree of disturbance to the optical mode which is undesirable and starts already appearing as can be appreciated by carefully inspecting IR image and brightness pattern. A thin oxide layer could be used as a spacer to carefully tune the scattering strength and find the best trade-off between these design parameters; this aspect will be focus of further studies. To conclude, tailoring EFSs shape to improve the scattering directionality could be considered for further performance boost, yet this would be require a very involved, tedious and less reproducible fabrication process that would greatly increase the complexity for producing these devices.

NOTE 2: DC DRIFT AND DATA ANALYSIS

Data acquisition and processing technique are introduced in the main text and follow the same approach as reported in [2, 3]. IR images are taken with our experimental setup for each value of applied DC voltage to the modulation region. Each picture corresponds to a snapshot of a different portion of the standing wave established within the waveguide, with pixel brightness being proportional to the wave intensity. After having recorded the full set of images, which correspond to the voltage range required to bring the initial standing wave sample from one EFS to the next one and a suitable voltage step (in this work we used $\Delta V = 100\,\mathrm{mV}$), they are stitched together to build up the complete and oversampled waveform. In case of multiple sampling regions, each probes a different standing wave portion, and then these are stitched together to reconstruct the extended interferogram.

Calibration of the device requires two steps. First, the scattering efficiency of each EFS needs to be measured as they may show slight differences that need to be accounted for. This is done by coupling a CW laser, for which the interferogram is known, and the brightness trace is normalised such that each peak and dip is at the same intensity (± 1). Incidentally, this procedure allows to extract a calibration curve that accounts for propagation loss as well, which for our waveguides is $<0.2\,\mathrm{dB/cm}$. Additionally, correction of chromatic-dispersion-induced distortions on the retrieved spectra is performed by using simulated dispersion curves.

Second, calibration of the electro-optic modulation needs to be performed and this is no trivial task due to its variability over time. As mentioned in the main text, lithium niobate is affected by the so-called DC drift effect. Bulk LN-based waveguiding systems (titanium indiffused or proton exchanged) include electrodes to drain the spatial charges out of the platform and remove the effect, this has however not been shown yet on LNOI. Annealing of the devices does help reducing or even eliminating the effect for short time (few weeks) and this allows us to conduct experiments and prove the operability of our devices. At low modulation speed, the shelf life of our spectrometers is still limited by DC drift which needs to be accounted for in data-processing.

During data analysis, we compensate for this effect by sweeping the modulation voltage over a wide range, beyond the value required to stitch interferograms between neighbouring EFSs, and calculate the average recorded periodicity using a CW laser of known frequency. The applied voltage corresponds to a physical distance on our sampling waveguide, thus

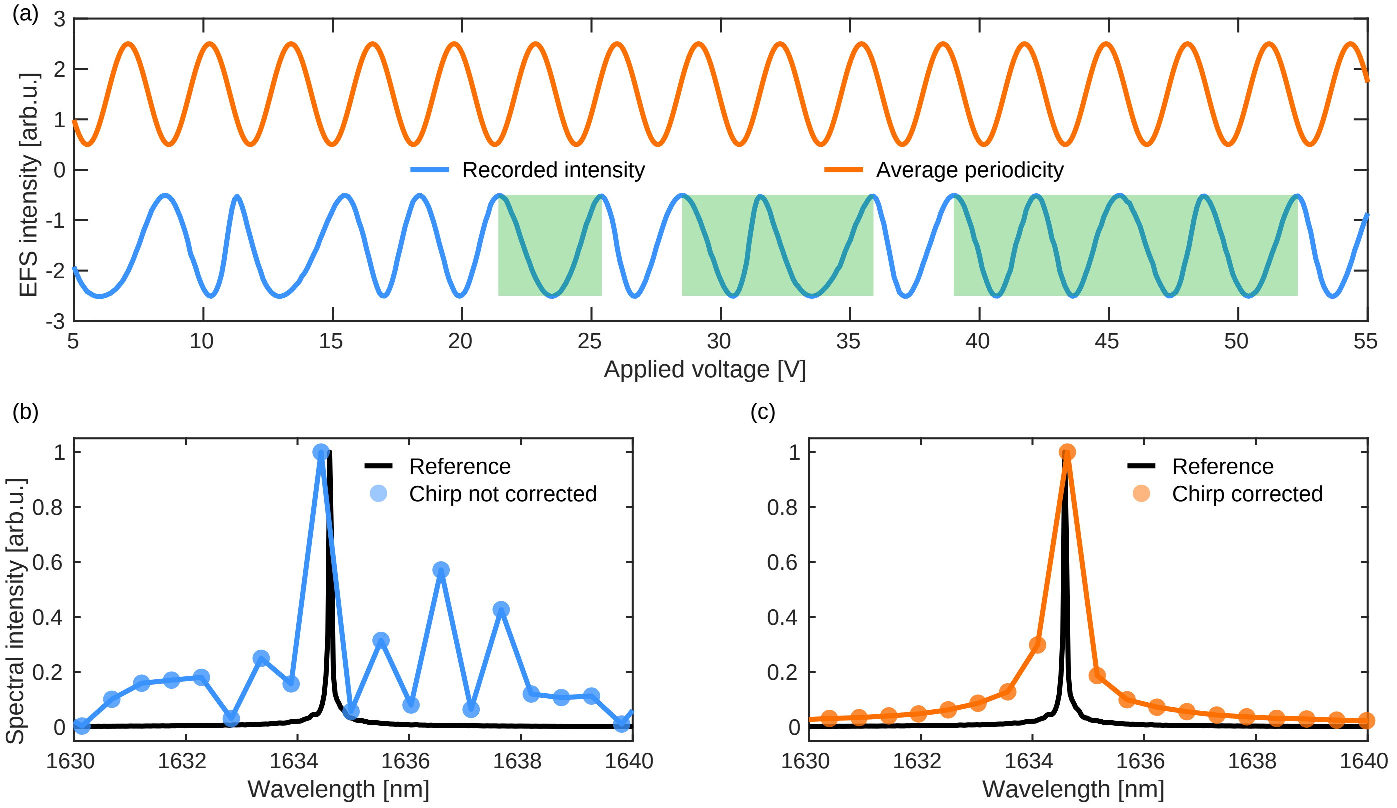

Figure S3: **Example of DC drift effect on the recorded interferogram.** CW laser at 1635 nm. (a) Recorded intensity for a single nanosampler versus applied voltage in raw form (light-blue line) and chirp corrected trace (orange line) by taking the average periodicity of the recorded intensity. The green patches highlight portions of the recorded trace where the measured periodicity is within a $\pm 5\%$ interval of the average pitch. (b) Retrieved spectrum for a non-corrected and (c) a chirp-corrected standing wave.

a nonlinear behaviour of the electro-optic effect causes a chirp in the interferogram. Figure S3(a) shows an example of recorded (light-blue line) and compensated (orange line) trace for a case of intense drifting effect for illustration purpose. The green patches highlight portions of the interferogram where the recorded periodicity is within a $\pm 5\%$ interval of the average pitch. Figure S3(b) and (c) instead show the reconstructed CW laser spectra at 1635 nm for a non-corrected and corrected chirping, respectively. The impact of DC drift is more deleterious for low coherence sources and neighbouring narrow spectral features, as seen in Fig. 4 of the main manuscript, effectively limiting the spectral resolution of our devices due to the appearance of spurious spectral features in form of sidebands. Relatively fast (hundreds of kHz/few MHz) modulation of the applied field, synchronised with the imaging system, would allow to neglect the effect as charges cannot follow the fast oscillations. We are currently working on speeding up the measurement and lower the exposure time of our imaging system, but nonetheless the problem would be solved by switching to fast integrated detectors (either electro-optical or micro-positioned array of InGaAs pixels).

NOTE 3: MEASUREMENT OF BROADBAND SOURCES

To demonstrate that our devices can be used to measure broadband spectra, we tested a spectrometer with resolution of $\Delta\lambda = 2.4\,\mathrm{nm}$ by coupling a 45.3 nm (FWHM) super-luminescent diode (SLED). Such a source has a coherence length of about 17 µm at the central wavelength of 1565 nm, which causes the interferogram to rapidly lose visibility beyond the main lobe. Nevertheless, we can resolve the interferogram and retrieve the source spectrum as illustrated in Fig. S4, where the inset shows analytical (light-blue line) and measured (orange line) interferograms over a reduced OPD window for illustration purposes. Spectral data are fitted to a Gaussian line-shape ($R^2 = 0.92$) from which the FWHM is computed and equals to $\delta\lambda = 47.9\,\mathrm{nm}$ (about 6% deviation from the value given by a commercial OSA). While most of the spectral information is contained in the interferogram portion close to the zOPD, non-perfect resolution of the interferogram wings cause artefacts to appear in the spectrum in form of spurious oscillations. This is due to the imaging system and normalisation procedure; it can be solved by employing a more sophisticated detection technique.

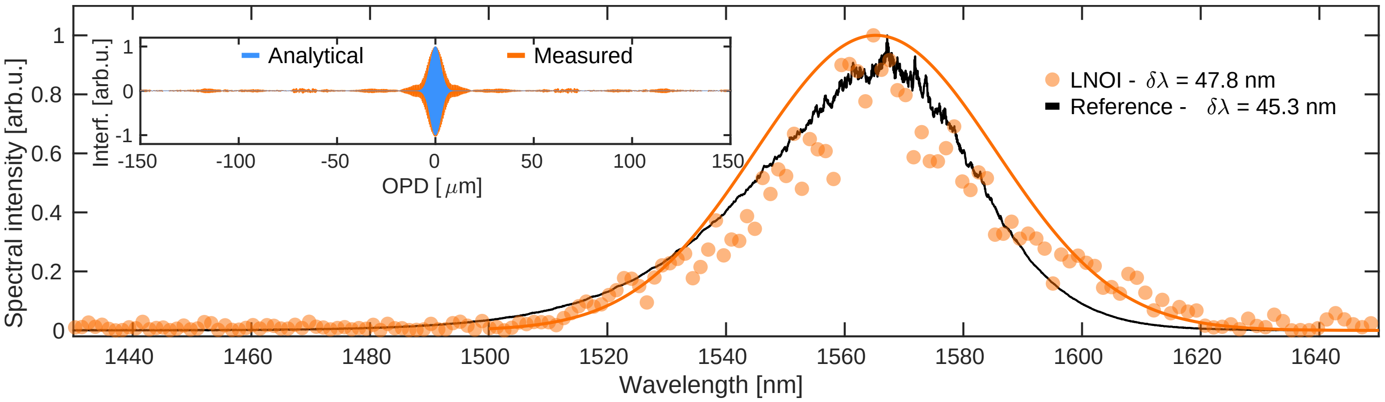

Figure S4: **Measured broadband source.** Super-luminescent diode spectrum at 1565 nm with calculated and measured interferograms displayed in the inset. Measured FWHM are $\delta\lambda = 45.3\,\mathrm{nm}$ (OSA) and 47.8 nm (our device). Data-points are fitted to a Gaussian line-shape giving an R^2 of 0.92 (orange line).

NOTE 4: BANDWIDTH LIMITATIONS AND MULTI-MODE OPERATION

As described in the main text and reported in [3], the bandwidth of our devices is solely limited by the single-mode condition of the designed waveguides, which according to numerical simulations starts around 1200 nm and extend to more than 2000 nm. It is important to mention, however, that the waveguides could be re-designed to operate at shorter wavelengths, thanks to the broad transparency of LN.

Given the extent of the single mode-spectral window, our available equipment sets an upper limit to the detectable wavelength at around 1700 nm due to the responsivity of InGaAs pixels cutting off in that range. Regarding the lower wavelength limit, even though our camera can detect shorter wavelengths, the waveguide is not single-mode any more below 1250 nm. Hence, it was not possible to prove a broader bandwidth with our equipment as we already pushed the tunable telecom lasers to their limit and used one butterfly laser at 1310 nm. Specifically, at 1064 nm the waveguide supports up to the third order TE mode, which has strong coupling to the fundamental TE mode, and this limits the bandwidth of the device. The measured bandwidth reported in Fig. 5 of the manuscript shall be thus interpreted as bandwidth of the experiment rather than of the device itself.

To support the above statements, we report in Fig. S5 the intensity trace obtained by monitoring a single scattering nanowire versus applied modulation voltage for two cases: multi-mode ($\lambda = 1064$ nm, blue line) and single-mode ($\lambda = 1635$ nm, orange line) operation. We strived to couple and reconstruct a CW laser spectrum at $\lambda = 1064$ nm with the same procedure, equipment and algorithm, however this was not feasible without substantially increasing the data analysis routine complexity. By inspecting the measured patterns it is clear that for a waveguide supporting multiple optical modes our procedure suffers of mode-mixing. Indeed, higher order modes having a lower effective index interfere by producing a standing wave of different periodicity with respect to the fundamental one. Overlaps between the interferograms results in a hard to interpret pattern that cannot be directly related to a CW laser interferogram. As anticipated in the main text, multi-mode operation would require complicated pattern recognition and deconvolution algorithms in order to separate the contributions of different optical modes, which is beyond the scope of this work.

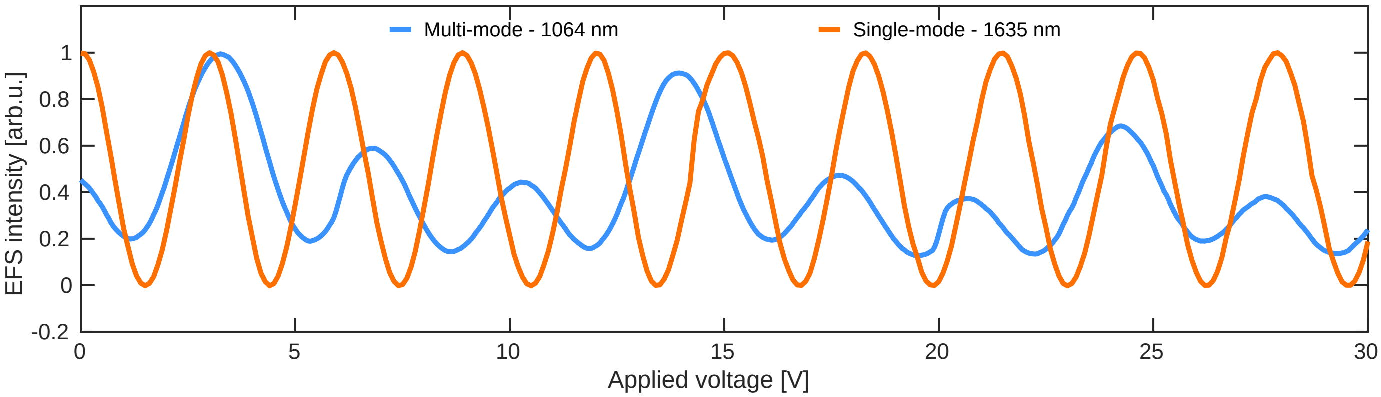

Figure S5: **Comparison of the interferogram trace.** Intensity versus applied modulation voltage for multi-mode ($\lambda = 1064$ nm, blue line) and single-mode ($\lambda = 1635$ nm, orange line) operation. The trace is obtained by monitoring the intensity profile from a single scattering nanowire and highlights the difficulty of reconstructing the CW interferogram in case of mode-mixing.

To further confirm this, Fig. S6 shows the simulated transverse-electric optical modes in our circuits at $\lambda = 1064$ nm, 1310 nm, 1550 nm and 1635 nm, from which it is clear how the waveguide supports up to the third order mode at $\lambda = 1064$ nm. Even though it is weakly guided, the TE_{03} mode has a large overlap with the fundamental one due to their same parity, which leads us to believe that mode-mixing is occurring in our waveguides and results in the interference pattern reported in Fig. S5. To conclude, the bottom panel of Fig. S6 reports the dispersion curve of the fundamental TE mode as obtained with our simulations, with the vertical dashed lines indicating the wavelengths corresponding to the reported mode-profiles.

Within the measurable bandwidth and in single-mode operation our devices perform consistently as discussed in the main text and illustrated in Fig. 5. To better clarify the seemingly increasing FWHM trend versus wavelength, we report in Fig. S7 a comparison between the CW laser peaks widths as obtained with our devices (left panel) and a commercial OSA (right panel). Fluctuations of the experimental data-points shall be once again attributed to the random nature of the DC drift effect that slightly influences the spectral width, besides its position, if not perfectly compensated for. The solid line shows a linear fit to the data, which is to be compared to the increasing trend measured with the OSA. The analysis confirms that the performance of our device, although overestimating the FWHM by roughly one order of magnitude, is consistent across the measurable bandwidth.

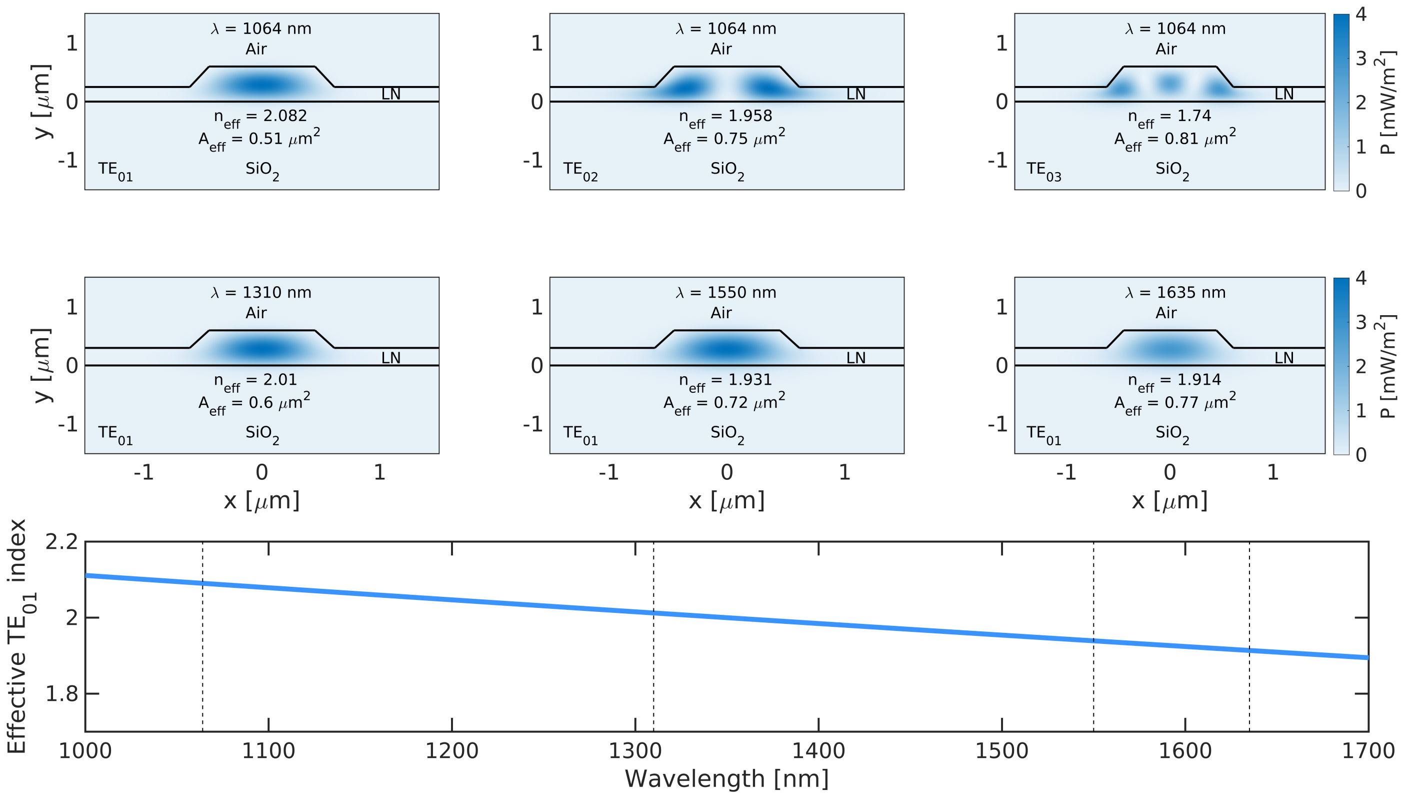

Figure S6: **Simulated transverse-electric optical modes.** Wavelengths of $\lambda = 1064$ nm, 1310 nm, 1550 nm and 1635 nm in our circuits. At 1064 nm the waveguide supports up to the third order mode (although weakly guided), which has a large overlap with the target fundamental TE mode. The single-mode window is calculated to start around 1250 nm. Effective indices and mode areas are reported in the respective panels, while the bottom plot shows the calculated dispersion curve of the fundamental TE mode versus wavelength.

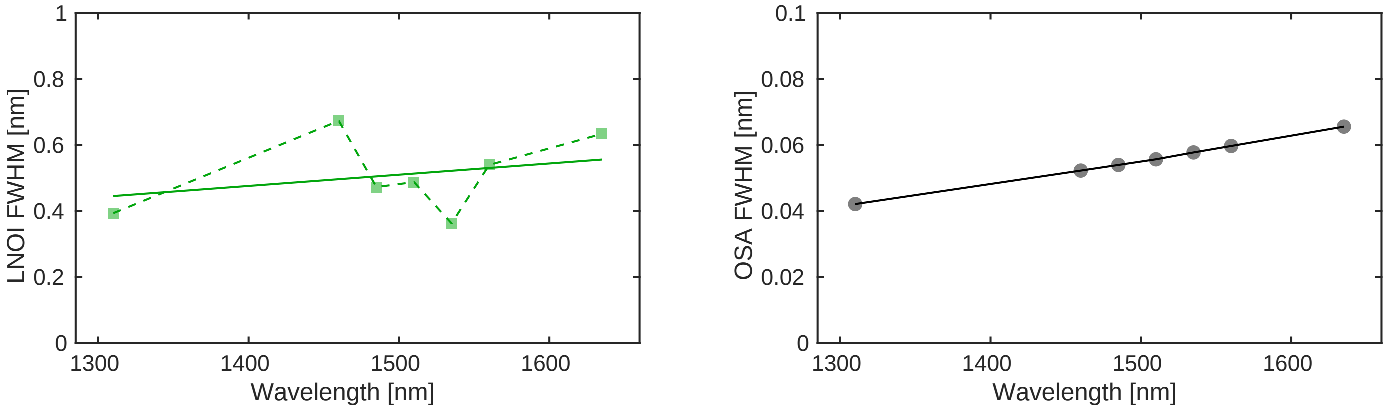

Figure S7: **Comparison of FWHM.** Tested CW laser spectra as reconstructed with our devices (left panel, solid line indicates a linear fit to the data) and measured with a commercial OSA (right panel).

REFERENCES

1. le Coarer, E. *et al.* Wavelength-Scale Stationary-Wave Integrated Fourier-transform Spectrometry. *Nature Photonics* **1,** 473–478. ISSN: 1749-4885, 1749-4893 (Aug. 2007).
2. Souza, M. C. M. M., Grieco, A., Frateschi, N. C. & Fainman, Y. Fourier Transform Spectrometer on Silicon with Thermo-Optic Non-Linearity and Dispersion Correction. *Nature Communications* **9,** 665. ISSN: 2041-1723 (Dec. 2018).
3. Pohl, D. *et al.* An Integrated Broadband Spectrometer on Thin-Film Lithium Niobate. *Nature Photonics* **14,** 24–29. ISSN: 1749-4885, 1749-4893 (Jan. 2020).
